# Feasibility of pulmonary MRI for nodule detection in comparison to computed tomography

**DOI:** 10.1186/s12880-020-00451-w

**Published:** 2020-05-20

**Authors:** Nan Yu, Chuangbo Yang, Guangming Ma, Shan Dang, Zhanli Ren, Shaoyu Wang, Yong Yu

**Affiliations:** 1 Department of Radiology, The affiliated hospital of Chinese traditional medical university, Xian Yang China, -2# Weiyang Western Road, Xian Yang, 712000 China; 2 Department of Medical Technology, The affiliated hospital of Chinese traditional medical university, Xian Yang, China

**Keywords:** Pulmonary nodules, Lung, Computed tomography, Magnetic resonance imaging, Radial VIBE

## Abstract

**Background:**

To assess the feasibility of various magnetic resonance imaging (MRI) sequences for the detection of pulmonary nodules by comparing the detection rate of computed tomography (CT).

**Methods:**

Forty-two patients with pulmonary nodules detected by multi-slice CT (MSCT) were prospectively enrolled in the present study between November 2016 and February 2017. Chest MRI was acquired within 24 h of CT. The MRI protocol included free-breathing radial VIBE (r-VIBE) and a conventional breathhold T1-weighted VIBE (C-VIBE) were analyzed by two independent radiologists. Both detection and morphology results of each MRI image were recorded. Subjective image evaluation in terms of overall nodule morphology on the MRI images was carried out using the 4-point scoring criteria. The MRI results were compared with those from CT, with the results of MSCT serving as the reference standard.

**Results:**

Two hundred and fifty-eight solid pulmonary nodules in 42 patients were detected by CT. The r-VIBE correctly detected 94% of the pulmonary nodules as compared with CT. The detection rate increased to 100% for lesions ≥6 mm. The C-VIBE had a lower overall detection rate (64.3%) of pulmonary nodules. The difference in the subjective image evaluation scores between the two sequences was statistically significant (*p* < 0.001).

**Conclusion:**

Significantly increased detection rates were obtained with free-breathing r-VIBE as compared with C-VIBE for the detection of pulmonary nodules and also provided more information when evaluating the nodules as compared with C-VIBE.

## Background

Given the improved soft tissue contrast and the lack of ionizing radiation, magnetic resonance imaging (MRI) is considered an efficient diagnostic tool for pulmonary lesions [1–4]. A number of different sequences have been reported for imaging the lung, including T2-weighted (T2W) half-Fourier single-shot turbo spin echo (HASTE) [5], T1-weighted (T1W) 3D gradient recall echo (GRE) with breath-hold [6], and ultrashort echo time (UTE) imaging [7]. A previous study [8] reports that MRI is suitable for lung screening, with excellent sensitivity, specificity, positive predictive value, and negative predictive value (more than 92%) for nodules ≥6 mm. For solid nodules, clinical studies show detection rates of 60 to 90% for lesions with a diameter of 5–8 mm, with the detection rate being close to 100% for lesions 8 mm or larger [9–11].

T1-weighted gradient echo sequences with volume volumetric interpolated breath-hold examination (T1 GRE VIBE) have been shown to allow for motion-free imaging during a single breath-hold. However, this sequence requires patients to hold their breath effectively, since respiratory motion artifacts can affect the evaluation of pulmonary nodules. The recently developed free-breathing radial VIBE (r-VIBE) sequence is reported to be less sensitive to motion as compared with the breath-hold Cartesian acquisition scheme in the conventional VIBE (C-VIBE) sequence. It can provide high-resolution images and is described as a valuable T1-weighted gradient-echo sequence for abdominal MRI in the examination of patients who are unable to hold their breath [12–17]. Thus, here, we assessed the role of r-VIBE in the detection of pulmonary nodules in comparison with breath-hold C-VIBE and MSCT.

## Methods

### Patient

The present study was approved by the institutional review board of our hospital, and all participating patients signed an informed consent. Patients with pulmonary nodules detected by MSCT were prospectively enrolled in the present study between November 2016 and February 2017. Patients were eligible to participate in the study if they were no contraindications to undergo MRI; MSCT showed at least one solid nodule sized between 2 and 30 mm; Patients underwent CT and MRI of the chest within 24 h. Exclusion criteria were younger than 18 years, suffering from respiratory problems such as dyspnea and/or irregularities, and contraindications to MRI such as pacemakers, metal implants, and severe claustrophobia.

No patient has contraindications for MRI. Seven patients were excluded from further analysis due to: being younger than 18 years old (*n* = 1); MRI imaging was acquired later than 24 h after MSCT (*n* = 3); or being unwilling to accept an MRI (*n* = 3). Thus, 42 patients were finally included in the present study (17 females and 25 males) with a mean age of 64.7 years old (age range: 25–87 years, mean age: 61.2 ± 10.3 years).

### Multi-slice computed tomography

CT scans were obtained using a 64-multidetector row scanner (Discovery CT 750 HD, GE Healthcare, USA) in end-inspirational breath-hold. No contrast medium was used. The scanning parameters were as following: 80/140 KV instantaneous switching of tube voltage, 260 mA tube current, rotation time: 0.5 s/rot, matrix: 512 × 512. Image reconstruction was performed in axial and coronal orientations using a 1.2-mm slice thickness.

### Magnetic resonance imaging

MRI studies were performed by a 3.0-T SKYRA MR scanner (MAGNETOM 3.0 T SKYRA MR scanner, Siemens Healthcare, Erlangen, Germany) using explorer gradients (maximum gradient of 40, 40, and 45 m T/m along the x, y, and z axis, respectively and a slew rate of 200 m T/m/ms) and a phased-array multi-coil system (12 elements). Images were obtained using broad spectrum non-contrast enhanced MRI sequences commonly applied for imaging lung lesions, including breath-hold C-VIBE and a r-VIBE sequence. The r-VIBE sequence was acquired using a totally free-breathing method without any navigator or trigger methods. Selected parameters of the applied sequences are shown in Table 1.

**Table 1:** Parameters of the applied MRI sequences

Parameter	r-VIBE (axial plane)	C- VIBE (axial plane)
TR (ms)	2.79	3.97
TE/TEs (ms)	1.39	1.29
Flip (st)	5	9
Turbo factor	–	–
SENSE factor	–	CAIPIRINHA
Plane	Axial	Axial
NSA	1	1
FOV (mm)	380	380
RecFOV (%)	100	81.3
Matrix	320 × 320	320 × 320
Slice thickness	1.2	3
Breath-hold	No	Yes
Acquisition time (s)	5:30	16``

### Image evaluation

Evaluation of the nodules was carried out by two independent radiologists with more than 5 years’ experience in chest radiology. In order to avoid detection bias, the procedure for observation is as follows. Firstly, radiologists were asked to record and mark all visible pulmonary nodules or lesions on MRI images. Discrepancies between the two radiologists in terms of lesion detection were resolved by consensus interpretation. Four different images can be reconstructed from a Dixon acquisition of C-VIBE: in-phase, out-of-phase, water signal-suppressed, and fat signal-suppressed images. The observer needs to read all the images and select the clearest image on which to mark the nodules. Secondly, the same two radiologists reviewed and record all visible pulmonary nodules or lesions on MSCT images 2 weeks after MRI images reading. Discrepancies between the two radiologists were resolved by consensus interpretation. Thirdly, the MRI findings were matched with MSCT by the same two radiologists, including marking and recording all visible pulmonary nodules or lesions both on MRI and MSCT, on MSCT but not on MRI, and on MRI but not on MSCT. The results of MSCT served as the reference standard, The size of positive lesions were also measured on MSCT. If disagreement occurred, the final results were given by a third reviewer with 20 years’ experience in chest radiology. All pulmonary nodules were assessed according to size, which was defined as the average of the longest and shortest diameters. The final diameter was obtained by averaging the results from the two radiologists. To assess nodule detection by MRI for each subgroup, nodules were classified as true positive (detected by both CT and MRI), false negative (not detected on MRI but shown on CT), and false positive (no evidence on CT but shown on MRI). The detection rate of each MRI sequence was defined as the proportion of nodules detected by both CT and MRI to all nodules identified in MSCT scans.

Subjective image evaluation in terms of overall overall subjective image on MRI images was carried out using the 4-point scoring criteria listed in Table 2, including the edge, internal structure, spicules, signs of lobulation, and pleural retraction and over all lung marking. Finally, the results of subjective image evaluation of the MRI were compared one by one with the MSCT images. Both the MSCT and MRI images were displayed on a dual-monitor diagnostic workstation at the same time.

**Table 2:** Image evaluation scoring criteria

	Image evaluation scoring criteria
1 = non-diagnostic	Severe blurred margins; internal heterogeneity cannot be seen
2 = poor	Moderate to severe blurred margins; internal heterogeneity cannot be delineated
3 = sufficient	Acceptable margins resulting in an image with clear signs of speculation, lobulation, and pleural retraction; and internal heterogeneity can be seen
4 = good	Sharp margins; lesion can be seen with good delineation of signs (including speculation, lobulation, and pleural retraction); internal heterogeneity is well delineated

### Statistical analysis

The diagnostic accuracy of each MRI sequence was defined as the proportion of nodules detected by both CT and MRI to all nodules identified in MSCT scans. A Mann-Whitney U-test was used to compare the subjective profile displayed score between MRI and CT. The agreement of the two radiologists with respect to the subjective profile displayed score for each nodule was assessed using Cohen’s kappa test, by comparing the percentage of nodule classified in each point scoring criteria. The data are expressed as the mean ± standard deviation. For all statistical analyses, a *p*-value less than 0.05 is considered significant. All statistical analyses were performed using SPSS 17.0 (SPSS, version 17.0; Chicago, IL).

## Results

### Lesion detection

A total of 258 solid pulmonary nodules were detected by MSCT. The lesion sizes were classified according to the Fleischner Society. There were 104 (40.3%) solid nodules with a maximum diameter greater than 6 mm; 87 (33.7%) with a diameter between 4 and 6 mm; and 67 (25.9%) with a diameter less than 4 mm. Four calcified nodules were detected on MSCT (the maximum diameters were 9.1, 6, 3.1, and 2.8 mm). One ground-glass nodule, 12 mm in size, was found on MSCT. Table 3 shows the results of solid nodule detection in both MSCT and MRI.

**Table 3:** Nodules detected by MSCT and MRI

Size	MSCT- detected nodules	Detection rate	MRI		
			True positive	False negative	False positive
r-VIBE					
≥ 6 mm	104	100%	104	0	0
4–6 mm	87	93.1%	81	6	2
≤ 4 mm	67	86.5%	58	9	2
C-VIBE					
≥ 6 mm	104	84.6%	88	16	2
4–6 mm	87	62.0%	54	33	4
≤ 4 mm	67	35.8%	24	43	2

**Fig. 1:**
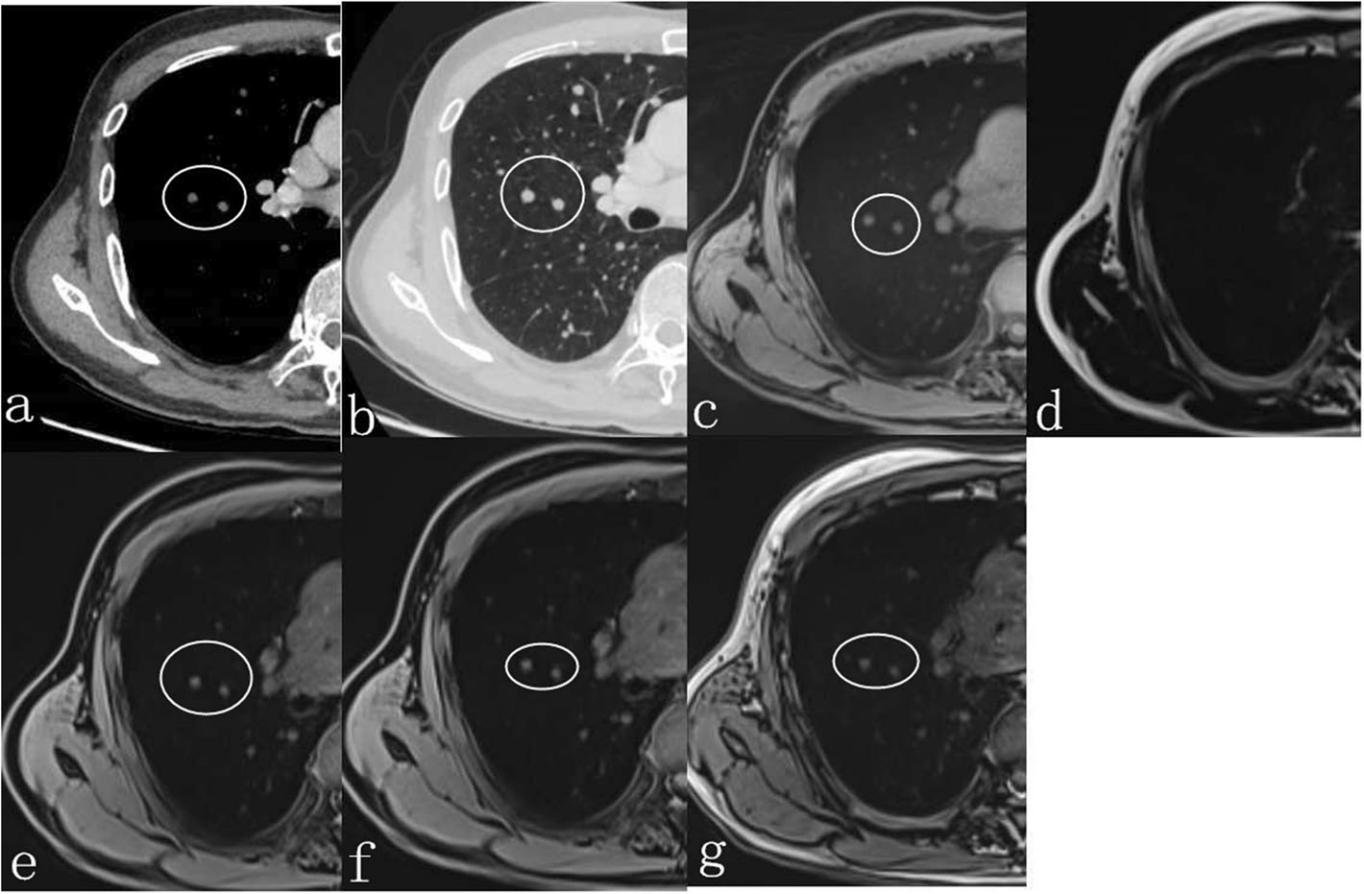
A 72-year-old female with two nodules (5.1 and 6 mm, white circle) on the right lung shown on MSCT (**a** and **b**). The nodules can be detected on r-VIBE (**c**), water signal-suppressed C-VIBE (**e**), in-phase C-VIBE images (**f**) and out-of-phase C-VIBE images (**g**), but cannot be detected on fat signal-suppressed C-VIBE (**d**)

**Fig. 2:**
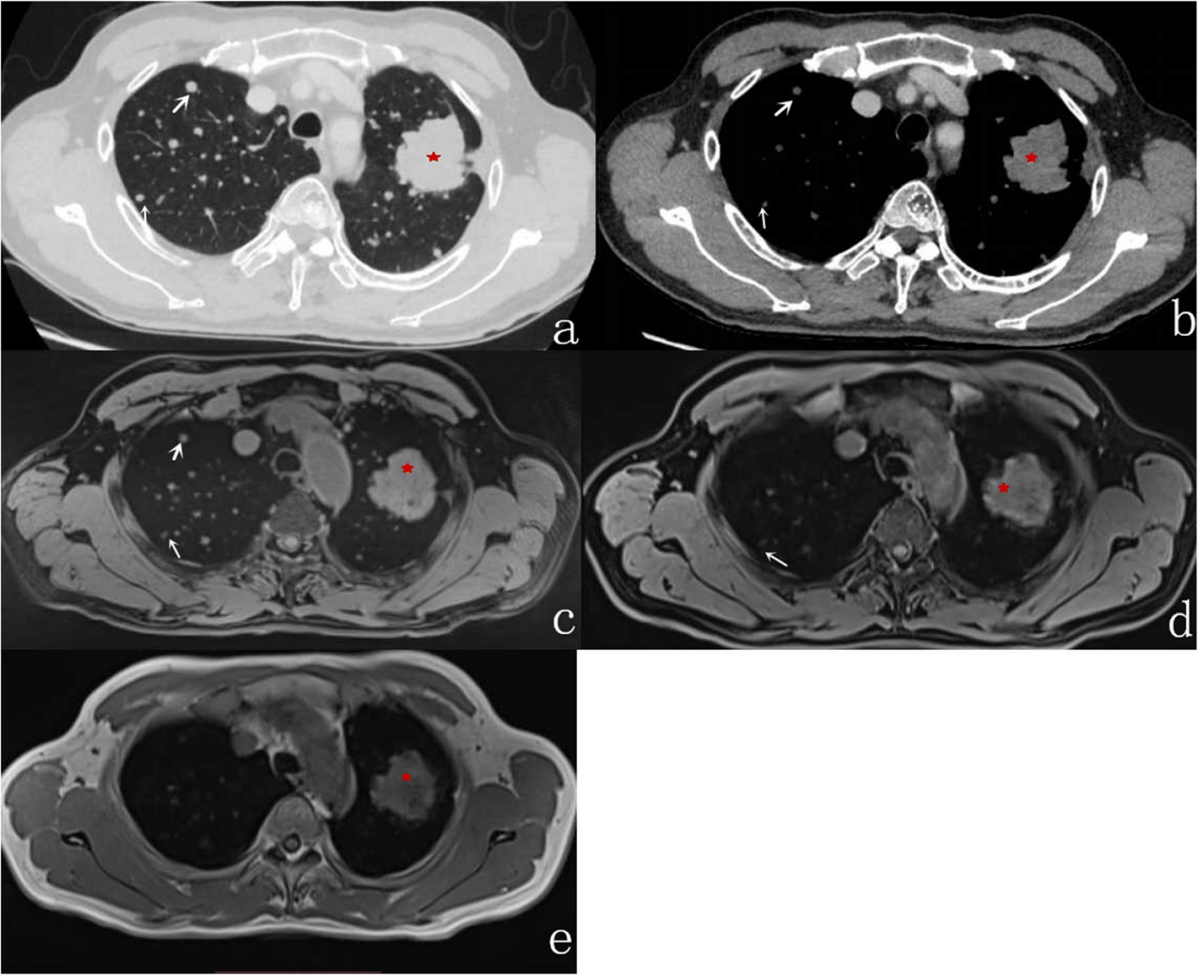
A 65-year-old male with lung adenocarcinoma and pulmonary metastasis. MSCT demonstrates a nodule (star) with pleural indentation (**a** and **b**):. r-VIBE imaging shows the nodule with clear boundaries (**c**):; however, it is not well depicted on water signal-suppressed C-VIBE (**d**) or in-phase C-VIBE images (**e**). Multiple nodules in the lungs can also be seen (**a** and **b**), among which, nodules 3.2 mm (thick arrow) and 2.8 mm (fine arrow) in diameter are clear on r-VIBE (**c**), but unclear on C-VIBE images (**d** and **e**)

**Fig. 3:**
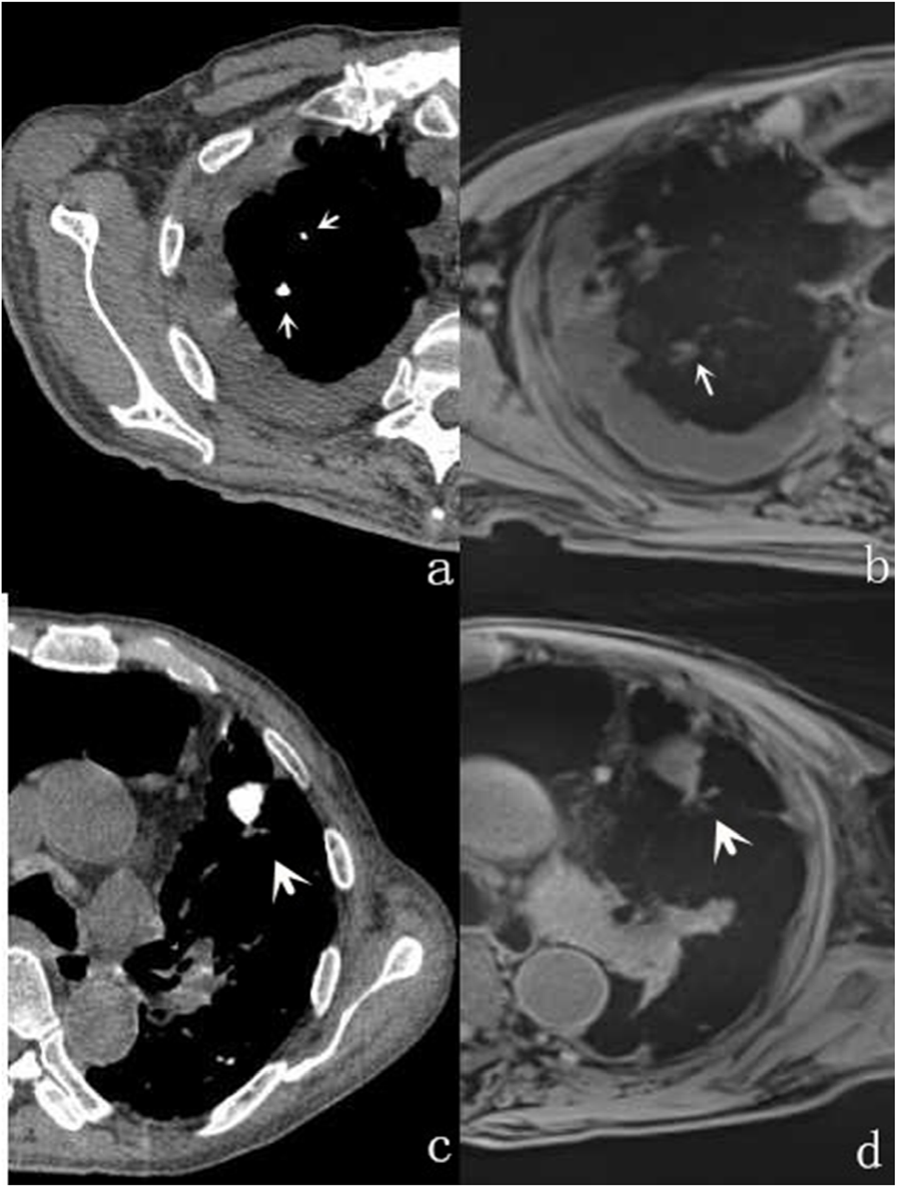
Two completely calcified small nodules shown on MSCT images (**a**) were both missed (**b**); a larger calcified 9 mm nodule can be seen on both MSCT (**c**) and r-VIBE (**d**)

**Fig. 4:**
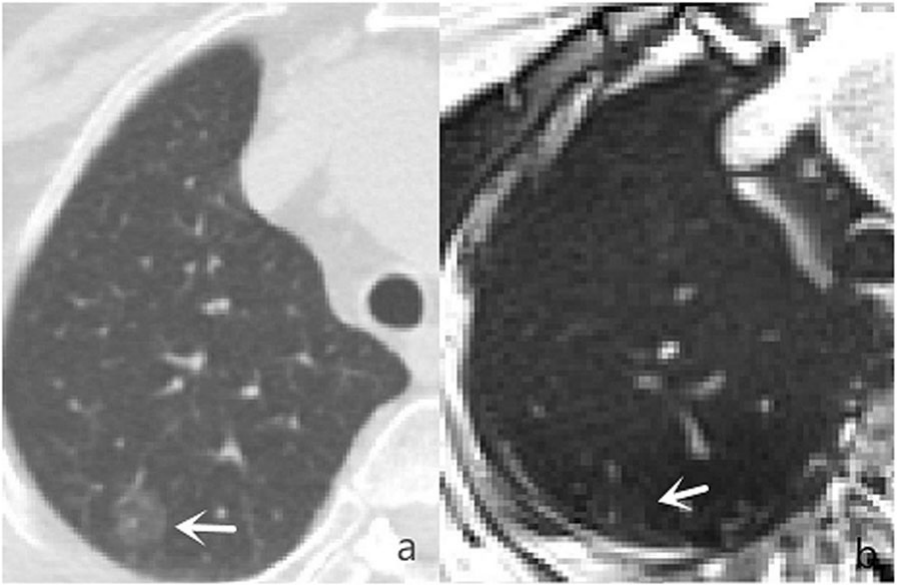
One ground-glass nodule 12 mm in size, was found in both MSCT (**a**) and r-VIBE (**b**)

In comparison with MSCT, the accuracy rate of r-VIBE in detecting pulmonary nodules was 94%. As summarized in Table 3, pulmonary nodules with a maximum diameter greater than 6 mm had a detection rate of 100% by r-VIBE. With respect to pulmonary nodules with a maximum diameter between 4 and 6 mm, the detection rate was 93.1% by r-VIBE (Fig. 1), and for nodules with a diameter less than 4 mm, the rate was 86.5% (Fig. 2). Of the 258 pulmonary nodules detected by MSCT, 15 were not detected by r-VIBE, of which 6 had a diameter of 4–6 mm (including 2 nodules located near the heart and 4 subpleural nodules) and 9 less than 4 mm (including 3 nodules located near the heart, 4 subpleural nodules, and 2 fully calcified nodules) (Fig. 3). The diameter of the smallest nodule detected by r-VIBE was 2.3 mm. Of the 243 nodules detected by r-VIBE, 4 (1.6%) were not detected by MSCT, all of which were in the section of pulmonary vessels. One ground-glass nodule, 12 mm in size, was found in both MSCT and r-VIBE (Fig. 4). C-VIBE had a lower detection rate (all over 64.3%) of pulmonary nodules in each subgroup. Regarding the 4 different C-VIBE images, in-phase and water signal-suppressed images were better for reviewing nodules than fat signal-suppressed and out-of-phase images (Fig. 1).

### Analysis of lesion profiles

The two radiologists had good agreement with respect to the subjective profile displayed score (kappa > 0.80). The scores given by observer 1 were analyzed. The morphological display of pulmonary nodules was evaluated in those larger than or equal to 6 mm. The nodules with a diameter less than 6 mm were excluded from further analysis due to the low detection accuracy of the C-VIBE sequence in those groups. The difference in the subjective profile displayed score between the two sequences was statistically significant (*p <* 0.001, Table 4)

**Table 4:** Subjective image evaluation of various MRI sequences for pulmonary nodules

Signs	Ability to display the morphology of nodules		*P*-value
	r-VIBE	C- VIBE	
Edge	3.6 ± 0.6	2.1 ± 1.2	0.00*
Internal heterogeneity	2.5 ± 0.3	1.5 ± 1.6	0.00*
Spicules	3.2 ± 1.5	1.5 ± 0.4	0.00*
Signs of lobulation	3.3 ± 1.8	1.1 ± 0.3	0.00*
Pleural retraction	3.8 ± 2.0	1.0 ± 0.8	0.00*
Lung marking	3.6 ± 2.1	1.2 ± 1.2	0.00*

*p < 0.05

.

## Discussion

The present study demonstrates the feasibility of the r-VIBE sequence as an alternative imaging technique for the detection of pulmonary nodules with high sensitivity and low false-positive rates. Solid nodules with a diameter greater than 6 mm were detected accurately by both C-VIBE and r-VIBE, with a sensitivity greater than 90%; however, the detection rate of nodules ranging in size from 4 to 6 mm was significantly different between C-VIBE (62.0%) and r-VIBE (93.1%).

The MRI sequence for pulmonary nodule detection demonstrated perfectly concordant results with those obtained by CT. The C-VIBE sequence with an ultra-short TE (< 1 ms) is reported to have the highest detection rate (69%) as compared with other sequences (T2 TSE, T2 SPIR, T2 STIR, T2 HASTE, and T1 out-of-phase) [14]. These results are similar to our findings showing a high detection rate (84.6%) with a threshold of ≥6 mm. Lungs can move upwards during free breathing, and in particular for patients with lung cancer, the use of C-VIBE depends on the ability of patients to hold their breath. Respiratory motion artifacts can cause significant blurring of small lesions, which limits diagnostic accuracy.

Conventional MRI sequences (C-VIBE) acquire k-space data in a line by line manner, which renders them sensitive to motion effects such as patient movement or blood flow [10]. Changing the way in which k-space is acquired and filled is an alternative method to overcome the sensitivity to motion [18]. Radial and spiral k-space sampling techniques have potential applications in imaging for lung cancer due to the management of respiratory motion. The r-VIBE sequence uses radial stack-of-stars acquisition, allowing a free-breathing T1W sequence that can aid accurate tumor volume identification and delineation due to its lower motion sensitivity. It uses rectilinear sampling in the z direction and radial sampling in the xy plane. In comparison with the conventional T1W VIBE sequence, which allows the entire thorax to be imaged during a 16-s breath-hold, r-VIBE sequences require a longer time for acquisition. However, without the breath-holding restrictions, it is possible to increase the spatial resolution. It is reported [19, 20] that r-VIBE images have significantly lower ratings of pixel graininess, which can be explained by the higher matrix and thinner slice thickness as compared with C-VIBE. Using a higher matrix enabled maximization of the definition of lesion structure. Additionally, due to the free-breathing sequence of r-VIBE, the higher matrix was easily added despite the prolonged acquisition time.

The r-VIBE is reported to have a similar image quality to C-VIBE on abdominal MRI. Chandarana et al., [15] reported higher scores for r-VIBE as compared with C-VIBE with respect to overall image quality, including hepatic edge sharpness, hepatic vessel clarity, and respiratory motion robustness. However, another study reported that r-VIBE has lower, but acceptable, scores as compared with C-VIBE on liver MRI [21]. In the present study, the morphological display of pulmonary nodules was also different between r-VIBE and C-VIBE. The r-VIBE was more valuable in displaying nodule profiles. Previous work on pulmonary nodules reports that radial VIBE with thickness of 2.5 mm and PET data acquired simultaneously using PET/MR imaging have high sensitivity for the detection of FDG avid nodules that are 0.5 cm in diameter or larger (88.6%) and low sensitivity for non-FDG avid nodules [22]. We reported higher sensitivity of nodule detection (100% for nodule larger than 6 mm) with r-VIBE with thickness of 1.2 mm.

It must be noted, however, that there were a number of false-negative diagnoses generated by r-VIBE. The percentage of false-negative findings depends on several factors, including the location and size of the nodules. Nodules near the heart and subpleural nodules are easily affected by movement; therefore, missed detection occurs easily. Nodule characteristics can also have an impact on the final results. Four calcified nodules were detected on MSCT (9.1, 6, 3.1, and 2.3 mm). The nodules with maximum diameters of 9.1 mm and 6 mm were detected by r-VIBE, while nodules with maximum diameters of 3.1 and 2.8 mm were missed.

The present study has certain limitations. Firstly, only the lung nodules detected by MSCT were used as the reference standard; therefore, there were no ‘true negative’ findings in our study. To avoid ambiguity, small nodule classification was dependent on the size of the nodules measured on CT but not on MRI; thus, there is a lack of data regarding the consistency in measurement results based on CT and MRI. Secondly, the difference in thickness affects the comparability of the detection rates of the two sequences; a thinner slice thickness of 1.2 mm was used for r-VIBE in the present study. However, it was not possible to complete a chest scan under breath-holding with a comparable slice thickness for C-VIBE. Thus, the thickness of the C-VIBE we collected was 3 mm covering the whole chest during one breath-hold. Of note, all the studies used an axial scan for MRI sequences to facilitate observation of nodules as compared with MSCT. However, coronal acquisition of C-VIBE would have allowed a thinner slice thickness still covering the whole chest during one breath-hold. Thirdly, only one ground-glass nodule was detected by MSCT, leading to a lack of assessment of the detection rate of such nodules in the present study. Fourthly, in the present study, the detection rate and display ability of pulmonary nodules in the two MRI sequences were compared; however, we did not consider the results of multi-sequence evaluation. Lastly, the parameters of the nodules, including size and location, were only evaluated by visual analysis. A further automated 3D volume analysis of pulmonary nodules is needed. Additionally, partly of nodule had unclear nature information due to small size to biopsy or unavailable follow up information..

## Conclusion

In conclusion, here, we established that pulmonary nodules with a maximum diameter greater than 4 mm could be detected by a free-breathing r-VIBE sequence. The r-VIBE sequence allows identification of pulmonary nodules with high sensitivity and low false-positive rates, and also provides more information regarding nodules than a routine C-VIBE sequence.

## Data Availability

The datasets during and/or analysed during the current study available from the. corresponding author on reasonable request.

## Abbreviations

MRI: Magnetic resonance imaging
CT: Computed tomography
MSCT: Multi-slice computed tomography
VIBE: T1-weighted gradient echo sequences with volume volumetric interpolated breath-hold examination
r-VIBE: Radial VIBE
C-VIBE: Cartesian acquisition scheme in the conventional VIBE
HASTE: Half-Fourier single-shot turbo spin echo ultrashort echo time
UTE: Ultrashort echo time
GRE: Gradient recall echo
T2W: T2-weighted
T1W: T1-weighted
